# Negative Regulation of Age-Related Developmental Leaf Senescence by the IAOx Pathway, PEN1, and PEN3

**DOI:** 10.3389/fpls.2019.01202

**Published:** 2019-10-08

**Authors:** Renee A. Crane, Marielle Cardénas Valdez, Nelly Castaneda, Charidan L. Jackson, Ciairra J. Riley, Islam Mostafa, Wenwen Kong, Shweta Chhajed, Sixue Chen, Judy A. Brusslan

**Affiliations:** ^1^Milken School, Los Angeles, CA, United States; ^2^Department of Biological Sciences, California State University, Long Beach, Long Beach, CA, United States; ^3^Department of Biology, Genetics Institute, University of Florida, Gainesville, FL, United States; ^4^Department of Pharmacognosy, Faculty of Pharmacy, Zagazig University, Zagazig, Egypt; ^5^Plant Molecular and Cellular Biology Program, University of Florida, Gainesville, FL, United States; ^6^Interdisciplinary Center for Biotechnology Research, University of Florida, Gainesville, FL, United States

**Keywords:** senescence (leaf), senescence accelerated, IAOx, glucosinolate, PEN1, PEN3, exosomes

## Abstract

Early age-related developmental senescence was observed in Arabidopsis *cyp79B2/cyp79B3* double mutants that cannot produce indole-3-acetaldoxime (IAOx), the precursor to indole glucosinolates (IGs), camalexin and auxin. The early senescence phenotype was not observed when senescence was induced by darkness. The *cyp79B2/cyp79B3* mutants had lower auxin levels, but did not display auxin-deficient phenotypes. Camalexin biosynthesis mutants senesced normally; however, IG transport and exosome-related *pen1/pen3* double mutants displayed early senescence. The early senescence in *pen1/pen3* mutants depended on salicylic acid and was not observed in *pen1* or *pen3* single mutants. Quantitation of IGs showed reduced levels *in cyp79B2/cyp79B3* mutants, but unchanged levels in *pen1/pen3*, even though both of these double mutants display early senescence. We discuss how these genetic data provide evidence that IAOx metabolites are playing a protective role in leaf senescence that is dependent on proper trafficking by PEN1 and PEN3, perhaps *via* the formation of exosomes.

## Introduction

Meristems continuously produce new organs, but most plants cannot sustain all these tissues. For this reason, net plant growth is a balance between the development and expansion of new organs and the senescence of older organs, mostly leaves. Senescence results in nutrients being transported from older leaves to newly developing organs (Bleecker and Patterson, 1997; Himelblau and Amasino, 2001; Fan et al., 2009; Davies and Gan, 2012; Schippers, 2015; Havé et al., 2017). Optimal transport of nutrients is essential for reproductive success; early senescence, often induced by abiotic stress, will result in inefficient transport of nutrients while late senescence can either increase yield or result in reduced nutrient transport likely due to increased leaf sink strength (Smart, 1994; Tollenaar and Wu, 1999; Ding et al., 2005; Derkx et al., 2012).

Numerous hormones, such as ethylene (Li et al., 2013), jasmonic acid (JA) (He et al., 2002), and salicylic acid (SA) (Morris et al., 2003), promote both defense responses and senescence (Guo and Gan, 2012; Jibran et al., 2013). Abscisic acid (ABA) is also a positive regulator of senescence (Liang et al., 2014; Yang et al., 2014). Q-DELLA mutants, which cannot repress GA responses, display early leaf senescence implicating gibberellic acid (GA) as another positive regulator of senescence (Chen et al., 2014), likely functioning upstream of JA, SA, and ethylene. The role of GA is likely more complex as increased expression of the GA catabolic enzyme, GA oxidase, occurs during senescence (van der Graaff et al., 2006). Brassinosteroids (BR) are also likely positive regulators of senescence since both BR biosynthesis and signaling mutants display delayed leaf senescence (Chory et al., 1994; Yin et al., 2002). The sole well-established phytohormone negative regulator of leaf senescence is cytokinin (Gan and Amasino, 1995; Kusaba et al., 2013).

Other positive regulators of senescence include members of the NAC and WRKY families of transcription factors (Hickman et al., 2013; Kusaba et al., 2013; Woo et al., 2013; Li et al., 2014; Garapati et al., 2015; Kim et al., 2018). These transcription factor families also function during defense responses (Nuruzzaman et al., 2013; Birkenbihl et al., 2017). Interestingly, a number of NAC and WRKY transcription factors slow down or attenuate the rate of senescence; these include VNI2, JUB, WRKY54, and WRKY70 (Yang et al., 2011; Besseau et al., 2012; Wu et al., 2012). The combined positive and negative regulations create a balanced rate of tissue breakdown and protein catabolism for optimal nutrient export.

The intersection between defense and senescence was noted with many transcriptomic analyses of leaf senescence (van der Graaff et al., 2006; Breeze et al., 2011). Numerous defense signaling molecules such as RLPs (Guo et al., 2004), RLKs, and TIR-LRR-NBS (Buchanan-Wollaston et al., 2005) are expressed in senescence and H_2_O_2_ levels increase during senescence and defense (Jajic et al., 2015). Although similarities exist, the molecular relationship between these two processes is not well understood. Our previous work identified *CYP79B2* as a senescence up-regulated gene that showed a parallel increase in H3K4me3 histone marks (Brusslan et al., 2015). Two redundant genes, *CYP79B2* (At4g39950) and *CYP79B3* (At2g22330), catalyze the aldoxime conversion of tryptophan into indole-3-acetaldoxime (IAOx) (Hull et al., 2000; Mikkelsen et al., 2000; Zhao et al., 2002). IAOx is a substrate for the phytoanticipin indole glucosinolates (IG), camalexin and other phytoalexins, and indole acetic acid (IAA, the chemical name for active auxin).

Glucosinolates prevent herbivory and fungal infection in the order Brassicales (Sønderby et al., 2010). Glucosinolates are amino-acid-derived thioglucosides, which are hydrolyzed by thioglucosidase myrosinases upon tissue damage, producing a wide variety of aglucone catabolites including isothiocyanates, thiocyanates, and nitriles. These toxic products are thought to play a defensive role (Bednarek, 2012) and are termed phytoanticipins since they are made in a non-toxic form, and can be quickly converted to toxic catabolites. Besides indole glucosinolates, benzyl glucosinolates (BG) are synthesized from phenylalanine with the first step being the production of phenylacetaldoxime by CYP79A2 (Wittstock and Halkier, 2002). In addition, aliphatic glucosinolates (AG) are produced after chain-elongation of methionine and conversion to the aldoxime form by CYP79F1 (Hansen et al., 2001) and CYP79F2 (Chen et al., 2003). IGs are important for innate immunity, mediating rapid callose production at papillae formed at the site of infection to block penetration by powdery mildew penetration pegs (Clay et al., 2009).

Camalexin acts as a antimicrobial and limits the growth of necrotrophic fungi and hemibiotrophic fungi and oomycetes (Stefanato et al., 2009; Bednarek, 2012). Camalexin induces programmed cell death in the necrotrophic fungus, *Botrytis cinerea* (Shlezinger et al., 2011). It decreases the fitness of a phloem-sucking insect, but does not affect a generalist insect. Camalexin synthesis is induced by JA, SA, ethylene, H_2_O_2_, and microbe-associated molecular patterns (Ahuja et al., 2012).

Auxin can be produced by one branch of the IAOx pathway and is reported to negatively regulate senescence through IAA29 interaction with WRKY57. IAA29 is degraded in the presence of auxin, allowing WRKY57 to negatively regulate JA-induced leaf senescence (Jiang et al., 2014).

**Figure 1:**
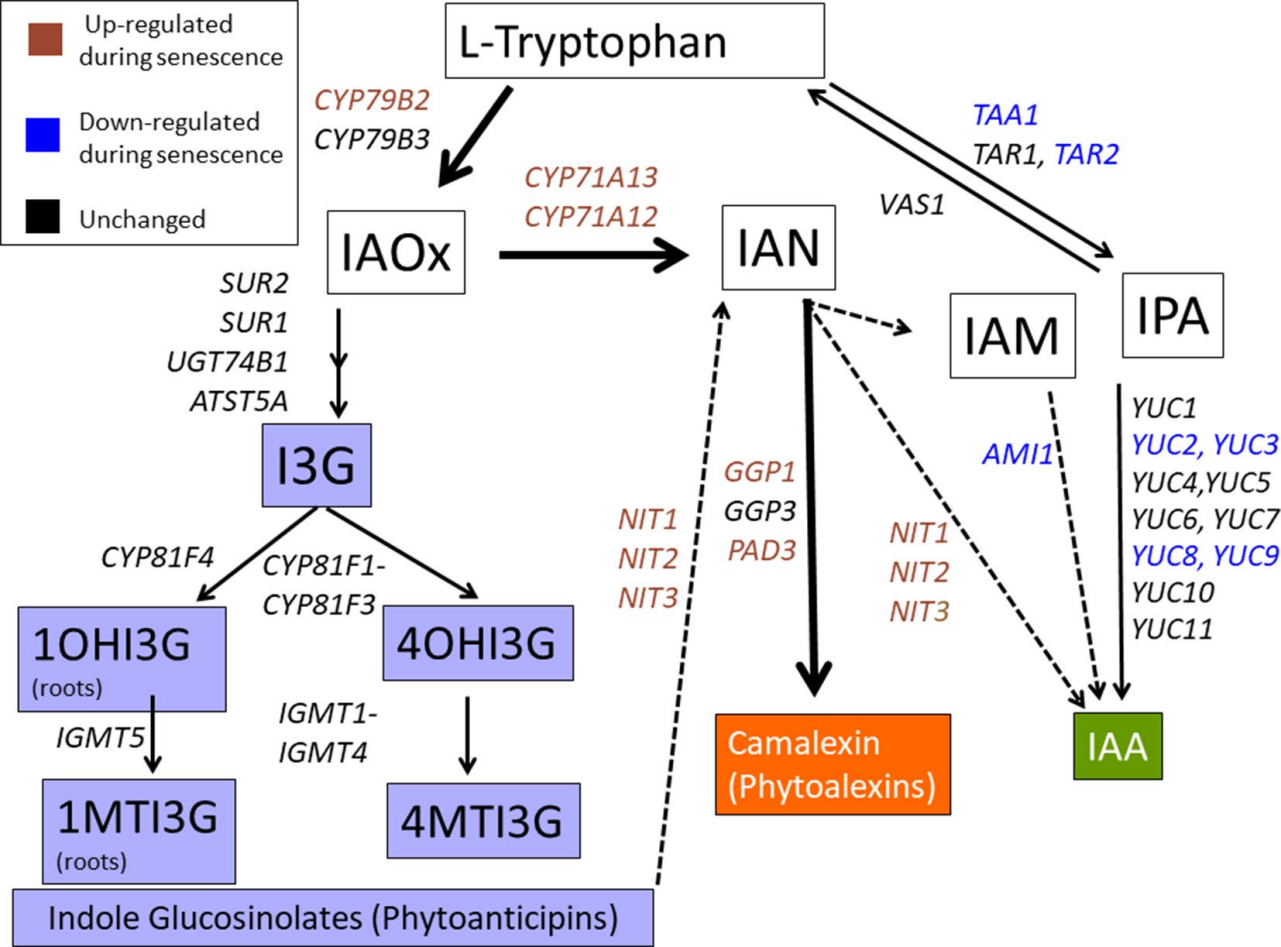
The IAOx pathway. CYP79B2 and CYP79B3 function just prior to the branch point between auxin (IAA), indole glucosinolates (IG), and camalexin biosynthesis. Solid lines represent established steps while dotted lines represent proposed steps. Colored text represents genes showing up or down trends as determined by microarray or RNA-seq (Breeze et al., 2011; Brusslan et al., 2015; Lyu et al., 2018). Gene expression in the phytoalexin (*de novo*) pathway is mostly up-regulated, gene expression in the IG phytoanticipin pathway is unchanged, while gene expression in the main IAA pathway is mostly down-regulated. I3G, 3-indolylmethylglucosinolate; 1OHI3G, 1-hydroxy-indolyl-3-methylglucosinolate; 4OHI3G, 4-hydroxy-indolyl-3-methylglucosinolate; 1MTI3G, 1-methoxy-3-indolylmethylglucosinolate; 4MTI3G, 4-methoxy-3-indolylmethylglucosinolate.

Microarray and RNA-seq data from leaf senescence time courses (GSE22982, GSE67777, and GSE97480) were scanned and increased expression of the camalexin pathway, unchanged expression of the IG pathway, and reduced expression of the IAA pathway were generally noted (**Figure 1**, **Supplemental Figure 1**). These findings prompted us to determine if the IAOx pathway was regulating leaf senescence. We discovered that *cyp79B2/cyp79B3* double mutants display early age-related developmental senescence, suggesting a protective role for IAOx metabolites. Mutants specifically affecting camalexin biosynthesis did not show early senescence, but a double *pen1/pen3* mutant did show early senescence. *PEN1* encodes a syntaxin involved in vesicle fusion with the plasma membrane while *PEN3* encodes an ABC transporter for PEN2 myrosinase-derived IG catabolites. IG levels were found to be greatly diminished in *cyp79B2/cyp79B3*, but unchanged in *pen1/pen3*. PEN1 and PEN3 are abundant proteins in exosomes, secreted extracellular vesicles that increase after SA treatment (Rutter and Innes, 2016). We propose that IAOx metabolite(s), which rely on PEN1 and PEN3 for correct localization, are playing a protective role during leaf senescence.

## Results

### IAOx Biosynthesis Mutants Display Early, Accelerated Leaf Senescence and Reduced Fitness

**Figure 2:**
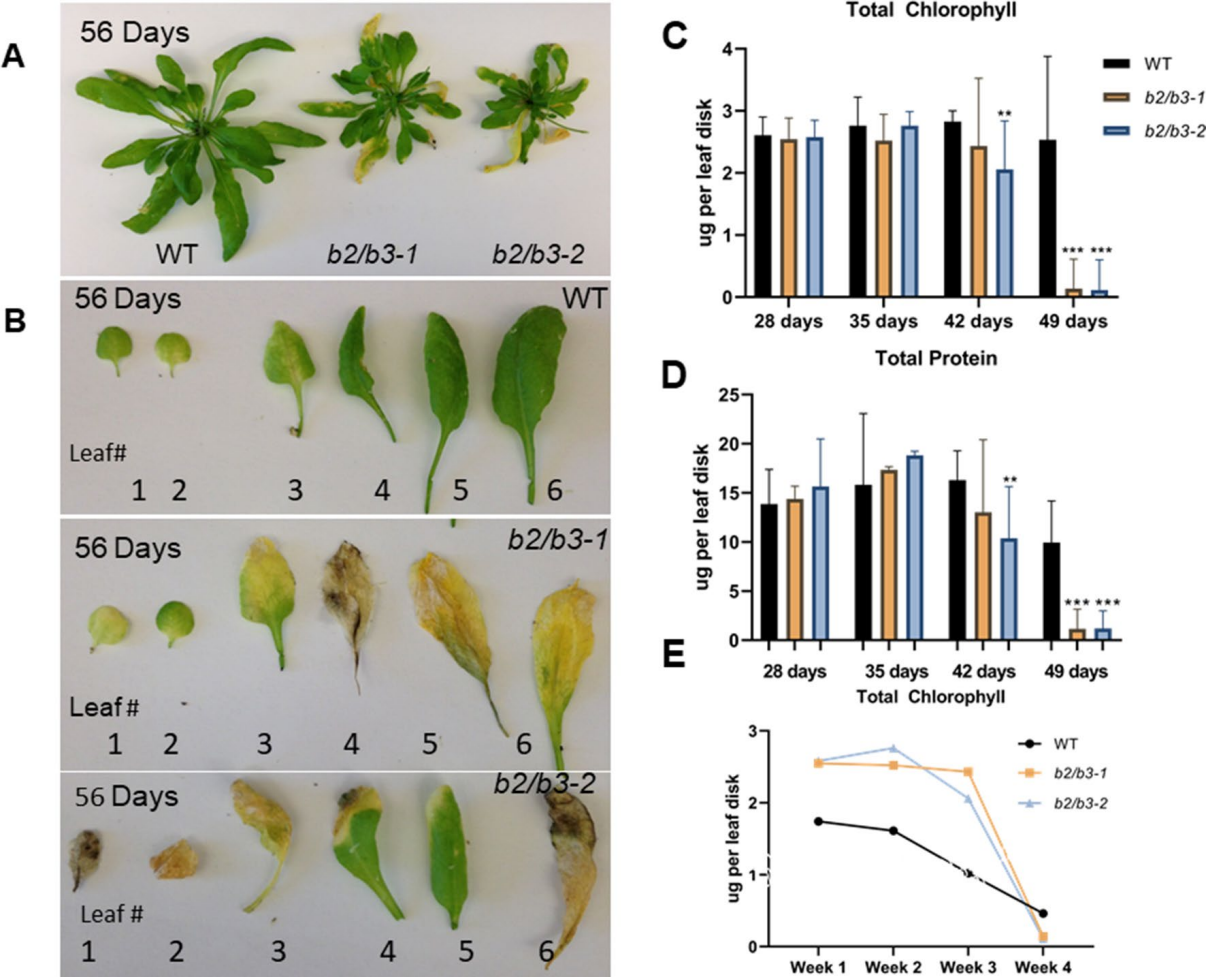
Early and rapid senescence phenotype of *cyp79B2/cyp79B3* double mutants. **(A)** Wild type Col-0 plants produce larger rosette leaves than double mutants (*b2/b3-1* and *b2/b3-2*). **(B)** Representative leaves 1–6 excised from plants and numbered in order of emergence. Total chlorophyll **(C** and **E)** and protein **(D)** levels per leaf disk were taken from leaf 6. Error bars represent 95% confidence interval, number of samples (*n*) = 3. Statistical analysis was done between WT and each *cyp79B2/cyp79B3* line at each time point (one-way ANOVA with Sidak’s multiple comparison test, **p < 0.01, ***p < 0.001). **(E)** Rate of chlorophyll loss during the last four time points. Days include 28, 35, 42, and 49 days for *cyp79B2/cyp79B3* lines (*n* = 3) and 49, 56, 63, and 70 days for WT (*n* = 6). Data presented are from one of three biological replicates.

*cyp79B2/cyp79B3* double mutants were constructed from single T-DNA insertion lines (**Supplemental Figure 2**). Two individual sibling double mutants were used throughout the study to avoid artifacts due to secondary T-DNA insertions (*cyp79B2/cyp79B3-1* and *cyp79B2/cyp79B3-2*). WT and *cyp79B2/cyp79B3* double mutant lines were grown in soil, and an early flowering phenotype (by approximately 3 days) was noted in the *cyp79B2/cyp79B3* lines as well as reduced sizes of fully expanded leaves 6 and 7 (**Supplemental Table 1**). After 8 weeks of growth, double mutants had a smaller overall rosette size (Zhao et al., 2002) and older rosette leaves were yellow (**Figure 2A**). Comparison of representative leaves 1–6 showed that WT leaves displayed some yellowing in leaves 1–3 while all six leaves showed reduced chlorophyll in the *cyp79B2/cyp79B3* lines (**Figure 2B**). Quantification of chlorophyll and total protein in leaf 6 showed significant reductions in both lines at 49 days (**Figures 2C, D**). The rate of chlorophyll loss was compared in WT and the *cyp79B2/cyp79B3* lines by plotting chlorophyll loss over a 4-week period. Both *cyp79B2/cyp79B3* lines showed a rapid loss of chlorophyll that spanned 1 week, while a more gradual loss spanning 2 weeks was observed in WT (**Figure 2E**).

**Figure 3:**
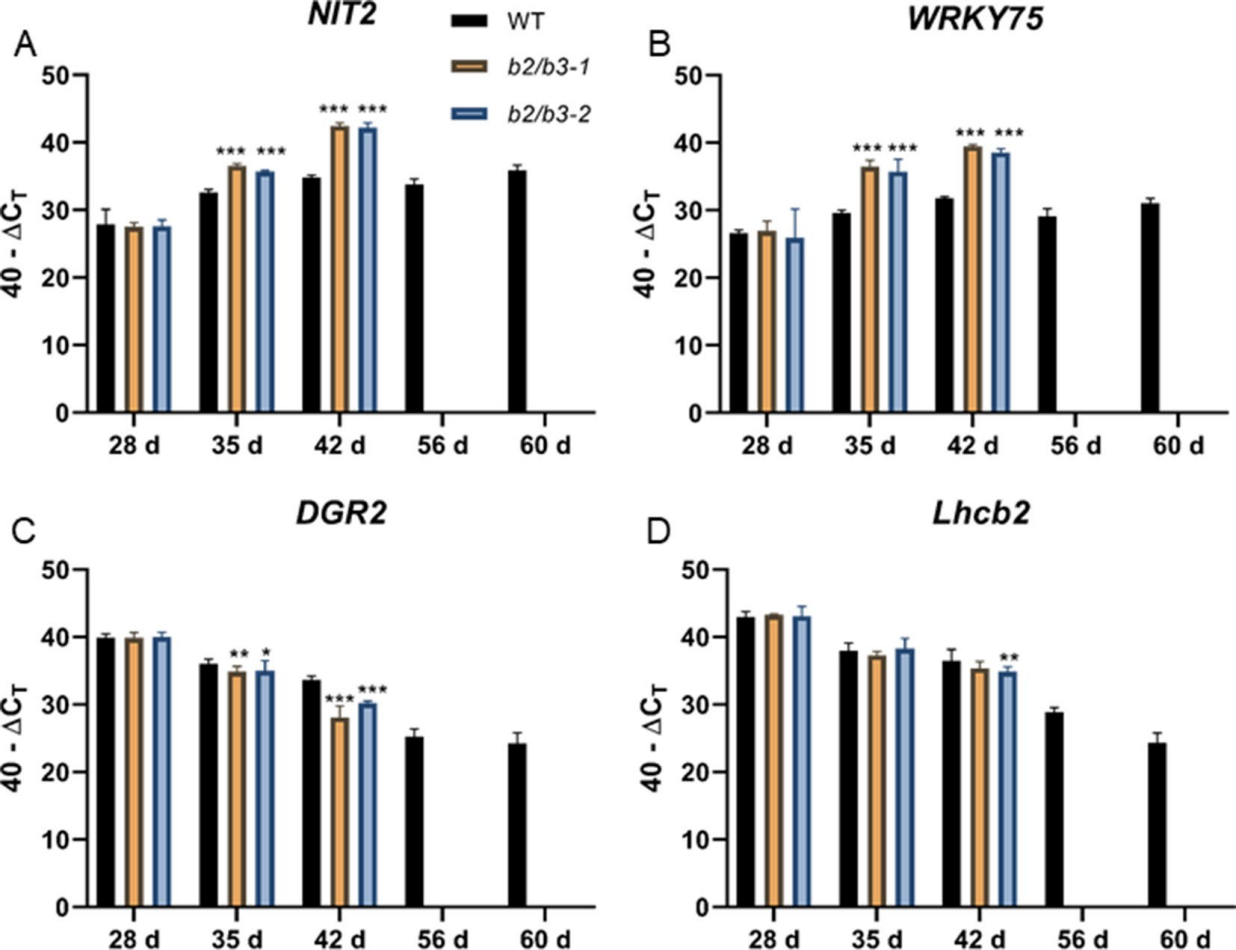
Gene expression in WT and *cyp79B2/cyp79B3* mutants. *NIT2* **(A)** and *WRKY75* **(B)** are up-regulated during senescence while *DGR2* **(C)** and *Lhcb2* **(D)** are down-regulated during senescence. RNA was extracted from leaf 6 at the indicated days. RNA could not be extracted from later time points for the *cyp79B2/cyp79B3* lines due to senescence. Error bars represent 95% confidence interval, *n* = 3. Statistical analysis was done between WT and each *cyp79B2/cyp79B3* line at each time point (one-way ANOVA with Sidak’s multiple comparison test, *p < 0.05, **p < 0.01, ***p < 0.001). Data presented are from one of three biological replicates.

Senescence-associated gene expression changes also occurred early in the *cyp79B2/cyp79B3* lines (**Figure 3**). This was seen for two SURGs: *WRKY75* and *NIT2*. These transcripts reached high levels in double mutant leaf 6 at day 35 and day 42; these levels were higher than those observed in WT at 60 days. One senescence down-regulated gene (SDRG), *DGR2*, displayed an earlier decrease in expression at day 42 in both *cyp79B2/cyp79B3* lines, but an earlier decrease was not observed in a second SDRG, *Lhcb2.4*. Accumulation of another marker of leaf senescence, H_2_O_2_, was approximated using DAB staining (Juszczak and Baier, 2014). H_2_O_2_ levels were increased in the *cyp79B2/cyp79B3* lines at day 49 (**Supplemental Figure 3**).

The early, accelerated leaf senescence phenotype was accompanied by early senescence of the inflorescence (**Supplemental Figure 4A**). Reduced fitness of these lines was shown by a significant decrease in dry bolt weight and total seed weight (**Supplemental Figures 4B, C**). The number of seeds per plant was also significantly reduced, but hundred seed weight, the number of seeds per silique, and germination rate were unaffected in the *cyp79B2/cyp79B3* lines (**Supplemental Table 2**). The diminished vegetative resources produced fewer inflorescences and seeds; however, these seeds were normal-sized and germinated at high efficiency. This is likely due to the strong sink strength of developing seeds, which prevents development of new floral meristems *via* global proliferative arrest (Wuest et al., 2016).

### Auxin Levels Are Reduced in IAOx Biosynthesis Mutants

**Figure 4:**
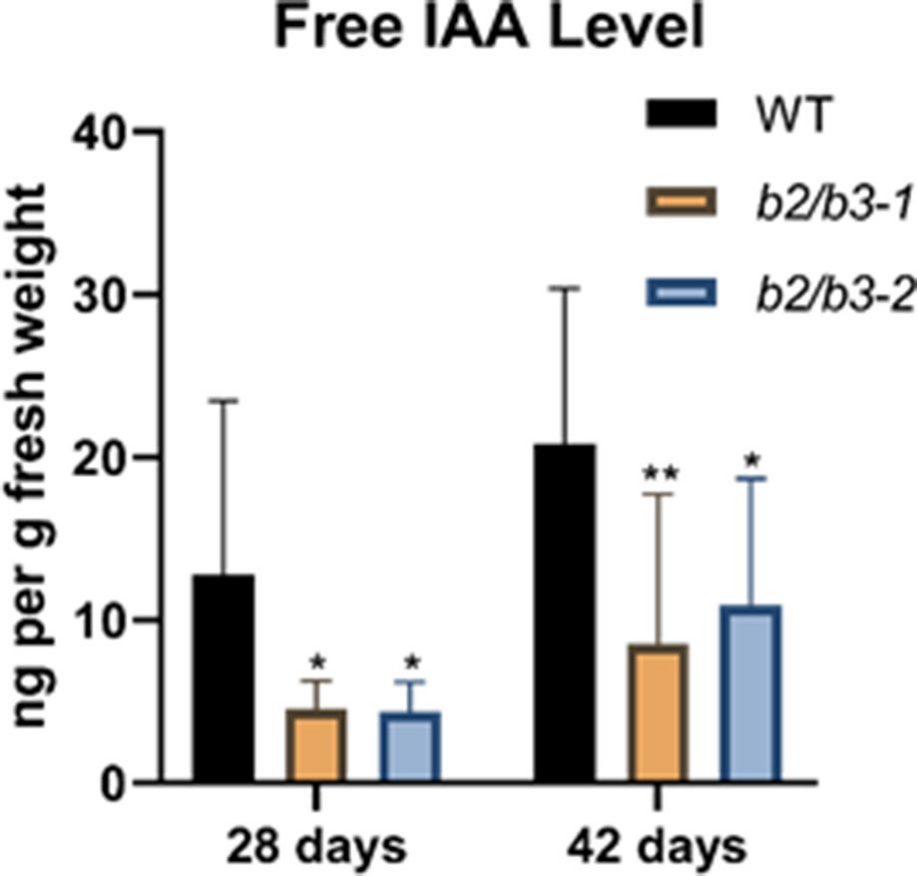
Endogenous IAA measurements. Endogenous free IAA was measured in leaf 7 at 28 and 42 days in WT and the two *cyp79B2/cyp79B3* double mutant lines. IAA in nanograms per gram of fresh weight is shown and error bars represent the 95% confidence interval, *n* = 3. Significant difference from WT at the same day are shown (one-way ANOVA, Sidak’s multiple comparison test, *p < 0.05, **p < 0.01). None of the IAA levels between days (for example: WT at 28 and 42 days) were significantly different. Data presented are from one of three biological replicates.

Endogenous non-conjugated IAA levels were measured using GC-MS/MS by the Plant Metabolomics Facility at the University of Minnesota (Barkawi et al., 2010; Liu et al., 2012). Free IAA levels were significantly reduced in both *cyp79B2/cyp79B3* lines at both 28 and 42 days (**Figure 4**), as expected since the IAOx pathway has been shown to contribute to IAA synthesis (Zhao et al., 2002; Zhao, 2014). A small, but not significantly different, increase in free IAA levels was noted in older leaves for all three lines, similar to a previous observation (Quirino et al., 1999). No other obvious auxin deficiency phenotypes, such as epinastic leaves, infertile flowers, or altered inflorescence architecture (Cheng et al., 2006; Stepanova et al., 2008), were noted, and thus it is unlikely that the approximately 50% decrease in free IAA levels observed in *cyp79B2/cyp79B3* lines is contributing to the dramatic early senescence phenotype. In addition, auxin-deficient lines do not show early senescence.

### Mutant Analysis Suggests IG Transport, But Not Camalexin, Plays a Protective Role During Age-Related Leaf Senescence

**Figure 5:**
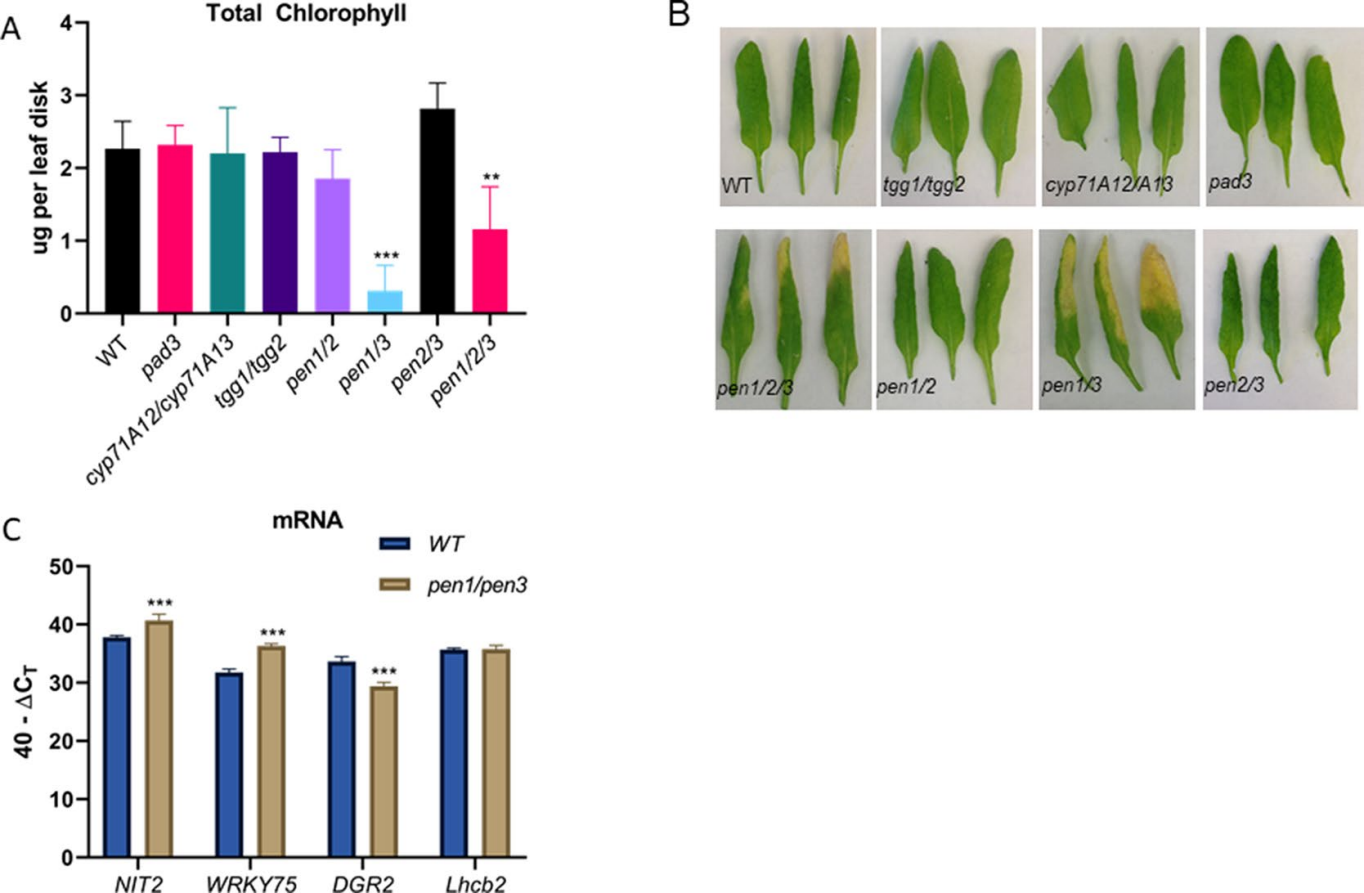
Total chlorophyll **(A)** levels per leaf disk taken from leaf 6 in camalexin biosynthesis mutants (*pad3*, *cyp71A12/cyp71A13*), in the IG myrosinase mutant (*tgg1/tgg2)*, and in *pen* mutants *(pen1/pen2, pen1/pen3, pen2/pen3* and *pen1/pen2/pen3*). Error bars represent 95% confidence interval, *n* = 6. Images of representative leaf 6 samples are shown in **(B)**. Expression of SURGs and SDRGs as determined by real-time qPCR **(C)**. RNA was isolated from leaves 6 and 7 at 38 days, *n* = 3. Error bars represent 95% confidence interval. Statistical analysis is shown in comparison to WT Col-0 (one-way ANOVA; Sidak’s multiple comparison test, **p < 0.01, ***p < 0.001). Data presented are from one of three biological replicates.

IAOx is a precursor for IGs, camalexin, and auxin. Mutant lines affecting IG and camalexin metabolism were obtained and verified (**Supplemental Figure 5**), and leaf senescence was tested in leaf 6 (**Figure 5**). Camalexin biosynthesis mutants (*pad3* and *cyp71A12/cyp71A13*) (Schuhegger et al., 2006; Nafisi et al., 2007) did not display early chlorophyll loss and leaf yellowing was not apparent, suggesting that blocking camalexin production does not result in early senescence (**Figures 5A, B**). The *tgg1/tgg2* myrosinase double mutants also displayed normal leaf senescence. TGG1 and TGG2 cleave glucose groups from IG molecules to form active catabolites upon tissue damage (Barth and Jander, 2006). In contrast, *pen1/pen3* double mutants and *pen1/pen2/pen3* triple mutants displayed early leaf senescence, which was accompanied by early changes in senescence-associated gene expression similar to those observed for the *cyp79B2/cyp79B3* lines (**Figure 5C**). *PEN1* encodes a syntaxin important for exocytosis of vesicles at the plasma membrane. The atypical PEN2 myrosinase catabolizes IGs, and these products are transported out of the cell by the PEN3 ABC transporter (Fuchs et al., 2016). The *pen1/pen2* and *pen2/pen3* double mutants do not show early senescence; it is only the *pen1/pen3* mutant combination that leads to early senescence. Early senescence was not seen in either *pen1* or *pen3* single mutants (**Supplemental Figure 5**). Both PEN1 and PEN3 are abundant in purified exosomes, as determined by proteomic analysis (Rutter and Innes, 2016). Exosomes are secreted vesicles associated with infection with biotrophic fungi (Hansen and Nielsen, 2017).

### Dark-Induced Senescence Is Unaffected in *cyp79b2/cyp79b3* and *pen1/pen3* Mutants

**Figure 6:**
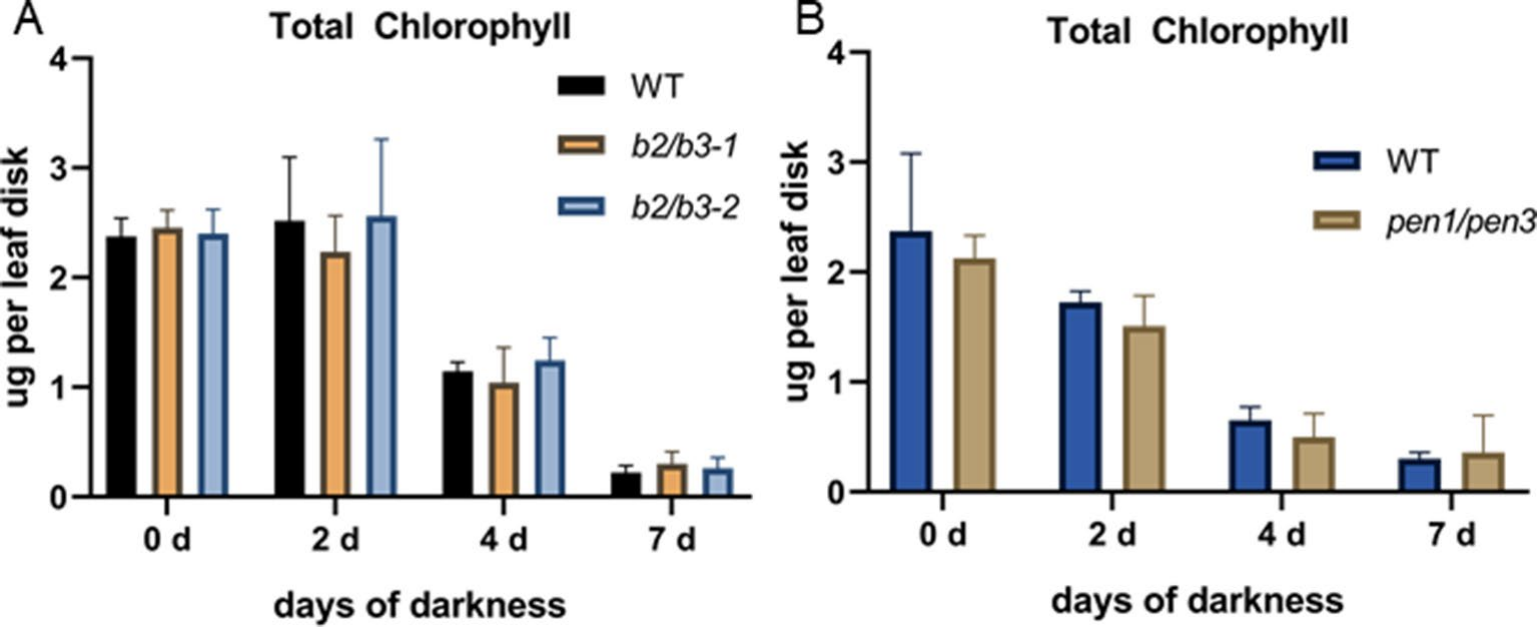
Dark-induced senescence in detached leaf 5. Total chlorophyll levels per leaf disk taken after dark incubation for the number of days indicated. WT and *cyp79B2/cyp79B3* are shown in **(A)** while WT and *pen1/pen3* are shown in **(B)**. Error bars represent 95% confidence interval, *n* = 4 for panel **(A)** and *n* = 5 for panel **(B)**. There were no significant differences between WT and mutant lines (one-way ANOVA, Sidak’s multiple comparison test). Data presented are from one of three biological replicates.

**Figure 7:**
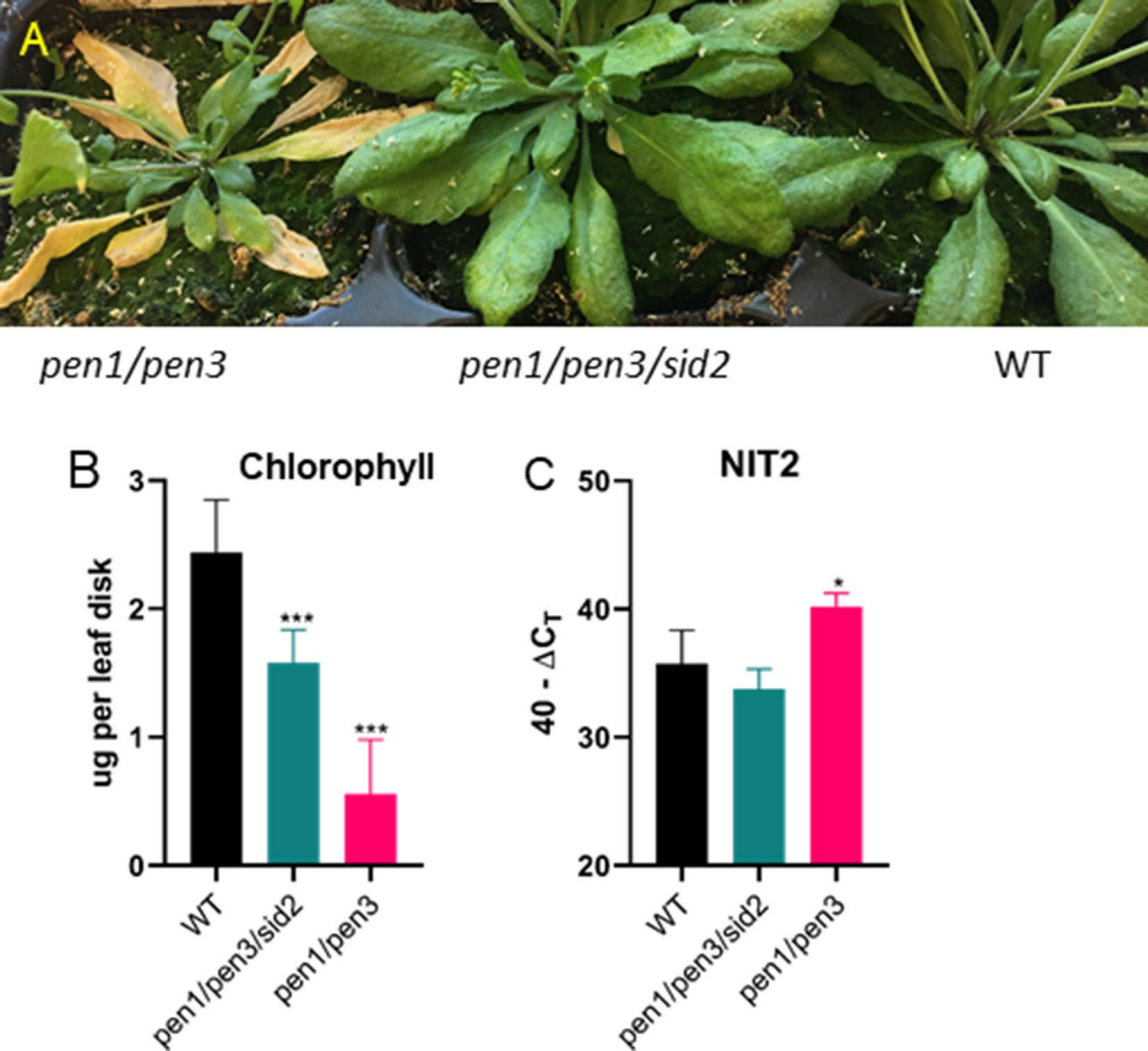
Early senescence in *pen1/pen3* is SA-dependent. **(A)** Forty-four-day-old plants were photographed, and early senescence is reversed by *sid2*, which blocks SA biosynthesis. **(B)** Chlorophyll (leaf 3, *n* = 6) and **(C)** *NIT2* gene expression (leaf 4, *n* = 3) were measured after 32 days of growth. Significant differences in comparison to WT are shown (one-way ANOVA, Sidak’s multiple comparison, *p< 0.05, ***p < 0.001). Data presented are from one of three biological replicates.

Senescence was induced by dark treatment of detached leaves, and chlorophyll loss was measured over 7 days. Chlorophyll loss was similar to WT for the *cyp79B2/cyp79B3* and *pen1/pen3* lines, demonstrating that early senescence in these lines is limited to age-related developmental leaf senescence (**Figure 6**). SA biosynthesis and signaling mutants also show a delay in developmental senescence, but not in dark-induced senescence (Buchanan-Wollaston et al., 2005). To determine whether SA was mediating the early senescence observed in the *pen1/pen3* mutant, a *pen1/pen3/sid2* triple mutant was constructed. *SID2* (At1g74710) encodes isochorismate synthase, and mutants fail to accumulate SA. The *pen1/pen3/sid2* triple mutant reversed the early senescence phenotype (**Figure 7**), demonstrating that early senescence is SA-dependent.

### IG Levels in *cyp79b2/cyp79b3* and *pen1/pen3* Mutants

**Figure 8:**
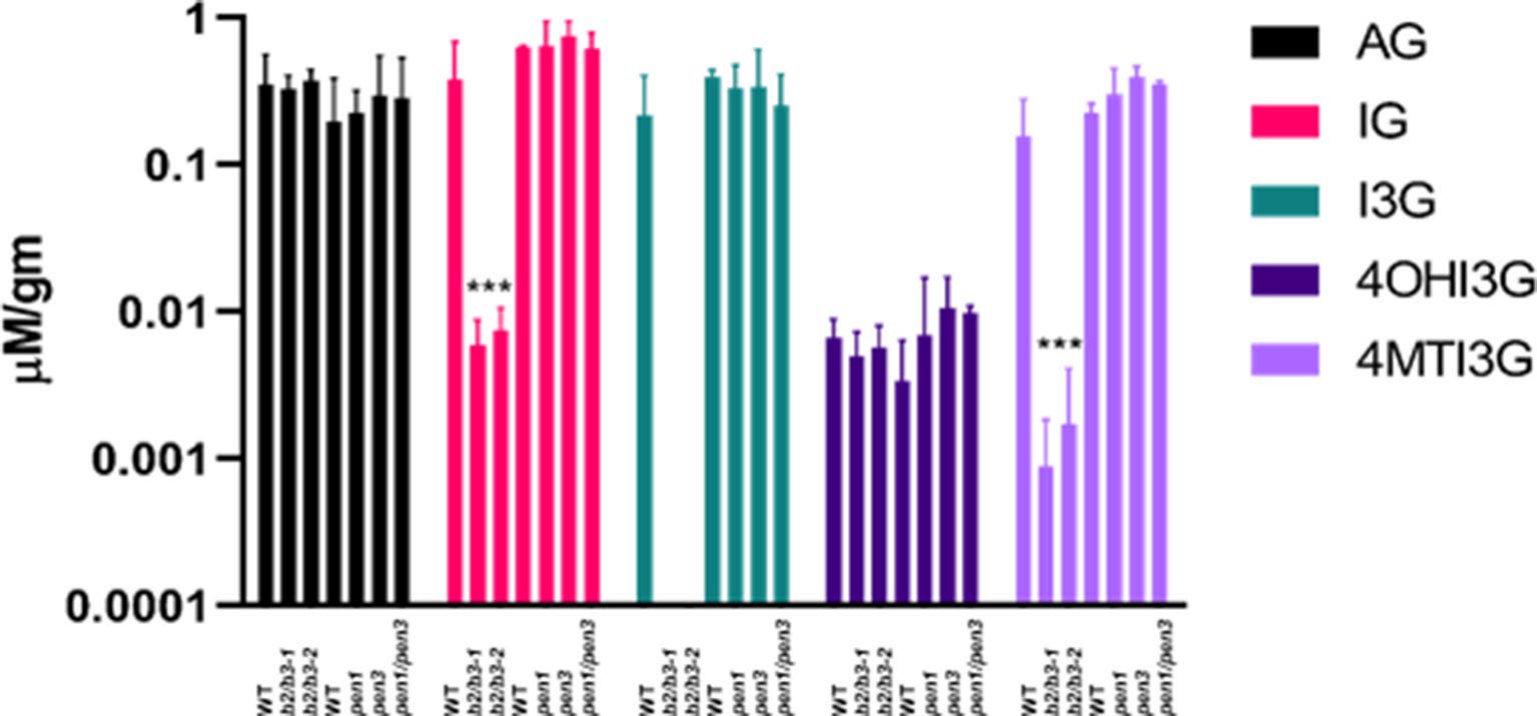
Glucosinolate levels in *cyp79B2/cyp79B3* and *pen* mutants. Leaves 6 and 7 were harvested from 42 day old plants, and glucosinolates were quantified. Total aliphatic glucosinolates (AG), total indole glucosinolates (IG), indole 3-glucosinolate (I3G), 4-hydroxyI3G (4OHI3G), and 4-methylI3G (4MTI3g). Significant differences to each experimental WT are shown for both *cyp79B2/cyp79B3* mutants. No significant differences were observed between WT and the *pen* mutants (one-way ANOVA, Sidak’s multiple comparison test, ***p < 0.001, *n* = 4 for *cyp79B2/cyp79B3* experiment and *n* = 3 for *pen* experiment). I3G was not detectable in *cyp79B2/cy79B3* mutants. Only leaf 6 was harvested for the *pen* experiment, which used *pen1-1*, *pen3-1*, and *pen1-1/pen3-1* alleles. Data presented are from three biological replicates.

Leaves were harvested from WT, *cyp79B2/cyp79B3*, and *pen* mutants at 42 days, and subjected to HPLC with a BG standard to quantify glucosinolates (Pang et al., 2009; Mostafa et al., 2016). IG levels were significantly decreased in both *cyp79B2/cyp79B3* lines, while AGs remained unchanged (**Figure 8**). Indolyl-3-methyl glucosinolate (I3G) was not detectable, but hyrdroxy-modified I3G, 4-hydroxy-indolyl-3-methylglucosinolate (4OHI3G) levels were unchanged. The methoxy-modified I3G, 4-methoxy-3-indolylmethylglucosinolate (4MTI3G) was significantly reduced in *cyp79B2/cyp79B3*. The complete loss of the precursor I3G at 42 days accompanied by normal levels of the downstream metabolite 4OHI3G suggests high CYP81F2 and CYP81F3 activity in *cyp79B2/cyp79B3* leaves; however, the significant reduction in 4MTI3G at 42 days suggests reduced IGMT1/IGMT2 anabolic activity and/or greater 4MTI3G catabolism in these same leaves. A reduction in total AG was observed in older leaves from all three lines (**Supplemental Figure 7**), which has been observed in previous studies (Brown et al., 2003). No significant reduction in total IG content was observed between 28 and 42 days in WT or in either of the *cyp79B2/cyp79B3* mutant lines.

Glucosinolates were also measured in leaf 6 harvested at 42 days from WT, *pen1-1*, *pen3-1*, and the *pen1/pen3* double mutant to determine if the reductions in IG metabolites observed in early senescing *cyp79B2/cy79B3* would also be observed in early senescing *pen1/pen3* (**Figure 8**). The levels of total AG and of I3G, 4OHI3G, and 4MTI3G did not change in the single or double *pen* mutants. IG-deficient and IG-replete lines both displayed early senescence, demonstrating that IG deficiency, on its own, cannot be regulating leaf senescence. Mutations in single members of IG biosynthesis gene families (*cyp81F1*, *cyp81F2*, *igmt1*, *igmt4*) and genes participating in the regulation of IG biosynthesis (*myc2* and *myb51*) (Schweizer et al., 2013; Frerigmann and Glgolashvlli, 2014) did not display early senescence, but IG levels were not quantified in these mutants (**Supplemental Figure 8**).

## Discussion

In the Brassicaceae, IAOx is produced from tryptophan and can be a precursor for auxin as well as two families of defense molecules: indole glucosinolate (phytoanticipins) and camalexin (phytoalexin). In Arabidopsis, IAOx biosynthesis mutants (*cyp79B2/cyp79B3*) show early leaf senescence with rapid loss of chlorophyll and protein accompanied by early SURG and SDRG expression changes. These data suggest that IAOx metabolites are attenuating the rate of leaf senescence. Mutants at various downstream pathways (**Figure 1**) were evaluated for early senescence phenotypes to determine which IAOx products were slowing down the rate of senescence. These included *cyp71A12/cyp71A13*, *pad3*, *tgg1/tgg2*, and various combinations of *pen* mutants. Although genes encoding the IAN branch of the pathway were up-regulated during leaf senescence, mutants in this pathway (*cyp71A12/cyp71A13* and *pad3*) did not senesce early. The only line that showed an early senescence phenotype similar to *cyp79B2/cyp79B3* was *pen1/pen3*. In addition, both of these double mutants showed normal dark-induced detached leaf senescence.

The PEN loci were first discovered in screens for Arabidopsis mutants with increased penetration by non-host biotrophic barley powdery mildew fungi (Collins et al., 2003). The PEN proteins play a role in localized secretion, activation, and transport of defense molecules (Fuchs et al., 2016). *PEN1* encodes a syntaxin, an integral plasma membrane protein that localizes PEN1-SNAP33-VAMP721/722 secretory SNARE ternary complexes to fungal entry sites for focused exocytosis (Kwon et al., 2008). These ternary complexes are proposed to function in secretion leading to callose deposition and the formation of papillae, paramural thickenings. *PEN3* encodes an ABC transporter (Stein et al., 2006) that can transport indole aglucones produced by the PEN2 glycosyl hydrolase (Lipka et al., 2005). PEN2 and PEN3 work together during fungal penetration resistance.

Interestingly, early and accelerated senescence was not observed in *pen1/pen2* double mutants; if PEN2 and PEN3 work in concert during leaf senescence, *pen1/pen2* and *pen1/pen3* should have the same phenotype. Yet, only *pen1/pen3* displays an early senescence phenotype. The differing phenotypes suggest that PEN2 is not required for production of the senescence-protective IAOx metabolite(s), but the metabolite(s) are likely to be transported by PEN3.

Both PEN1 and PEN3 are abundant components of small (150 nm diameter), extracellular vesicles called exosomes. In plants, these extracellular vesicles were noted at the site of cell wall thickening that serves as a barrier from penetration by an appressorial germ tube of biotrophic fungal pathogens (An et al., 2006b). Exosomes are released into the apoplast when multivesicular bodies (MVBs) fuse with the plasma membrane. Exosomes and MVBs were proposed to play a protective role during the hypersensitive response by occluding plasmodesmata and constraining ROS (An et al., 2006a). Encasements surrounding fungal haustoria selectively import the ternary SNARE complex (PEN1, SNAP33, and VAMP722) along with PEN3 and PMR4 glucan synthase. In this same study, the extended stability of GFP::PEN1 fluorescence in the acidic apoplast led to the suggestion that PEN1 is enclosed in an exosome (Meyer et al., 2009). Exosomes were purified from apoplast extracts of non-infected Arabidopsis leaves, and proteomic analysis showed high abundance of both PEN1 and PEN3. A number of antioxidant enzymes were also identified: glutathione-S-transferases, thioredoxins, ascorbate peroxidase, and catalase. In addition, ESM1, an epithiospecific modifier that favors isothiocyanate vs. nitrile formation from indole aglucones (Burow et al., 2008) is exosome resident. PEN3 and ESM1 presence suggests that IG catabolites reside in exosomes. More exosomes were observed after leaves were treated with SA (Rutter and Innes, 2016), and early senescence in *pen1/pen3* was found to be dependent on SA biosynthesis (**Figure 7**). We do not yet know whether exosomes can form in *pen1*, *pen3*, or *pen1/pen3* mutants, but it is possible that exosomes are contributing to leaf senescence.

Decreased auxin levels in the *cyp79B2/cyp79B3* mutant lines could also contribute to senescence. Reduced auxin signaling has been associated with an increased defense response leading to resistance to pathogen infection (Navarro et al., 2006) while increased auxin signaling is associated with virulent infection (Thilmony et al., 2006). Overexpression of the AFB1 auxin receptor reduced SA biosynthesis after *Pseudomonas syringae* infection (Robert-Seilaniantz et al., 2011), which would result in decreased resistance. Defense genes are up-regulated during senescence (Guo and Gan, 2012), and therefore it cannot be ruled out that reduced free auxin in *cyp79B2/cyp79B3* lines could contribute to up-regulation of defense and senescence by stimulating SA biosynthesis. A negative regulatory role of leaf senescence by auxin is contradicted by the small increase in free IAA levels seen in all three older leaf tissue samples (**Figure 4**) and in another study (Quirino et al., 1999). The input of the IAOx pathway to overall auxin biosynthesis is considered minor in comparison to the IPA pathway (Sugawara et al., 2009), and the strong developmental phenotypes associated with location-specific auxin loss (Zhao, 2014) were not observed in the *cyp79B2/cyp79B3* lines; as such, contributions to senescence by auxin made *via* the IAOx pathway are likely small.

Cleavage of the glucose moiety from glucosinolates is catalyzed by numerous myrosinases, including TGG1 and TGG2. Herbivores damage plant tissue, bringing glucosinolates (located in S cells near the phloem) and myrosinases (located in distinct cells also near the phloem) into direct contact (Koroleva et al., 2000). The resulting aglucones rearrange into toxic isothiocyanates or are diversified by specifier proteins into toxic nitriles, epithionitriles or thiocyanate, which deter further herbivory (Wittstock and Burow, 2010). *tgg1/tgg2* double mutants are unable to degrade AGs and sustain only low levels of IG degradation. The TGG1 and TGG2 myrosinases cannot be producing the senescence-protective metabolite(s) because normal senescence was observed in *tgg1/tgg2* mutants. AG levels decrease in older leaves of WT and the *tgg1/tgg2* double mutant, indicating that senescence-related AG degradation is not mediated by the TGG1 and TGG2 myrosinases (Barth and Jander, 2006). A similar decrease in total AGs in older leaves was observed in WT and the *cyp79B2/cyp79B3* double mutants (**Supplemental Figure 6**), suggesting that the resulting AG catabolites are not playing a protective role during leaf senescence.

Mutants that block early steps in the IG pathway (Bak et al., 2007) result in IAA overproduction and a severe phenotype, making evaluation of age-related senescence difficult. Later steps in the pathway are catalyzed by enzymes encoded by tandemly-arranged gene families. *CYP81F1*, *CYP81F3*, and *CYP81F4* are adjacent genes while *CYP81F2* is unlinked. *IGMT1*–*IGMT4* are tandemly arranged while *IGMT5* is unlinked (Pfalz et al., 2016). Based on senescence RNA-seq data (Brusslan et al., 2015), T-DNA insertions disrupting the most highly expressed gene family members (*CYP81F1*, *CYP91F2*, *IGMT1*, and *IGMT4*) were isolated, in hopes of reducing IG synthesis partially as to avoid IAA overproduction. Mutants disrupting single gene family members did not show an early senescence phenotype. In addition, T-DNA insertions disrupting positive regulators of IG synthesis (*myc2* and *myb51*) did not show early senescence (**Supplemental Figure 8**). I3G, 4OHI3G, and 4MTI3G were not measured in these mutant lines, but genetic redundancy likely maintained IG synthesis. Disruption of multiple family members by genome editing may be useful in producing a line with a specific reduction in IGs that does not overproduce IAA.

More severe lesions were observed in *cyp79B2/cyp79B3* mutants in comparison to WT after treatment with fumonisin B1 (FB1), a mycotoxin that elicits reactive oxygen species (ROS)-induced lesions. This observation suggests IAOx metabolites attenuate ROS-induced programmed cell death (Zhao et al., 2015). FB1-induced lesions were also more severe in *tgg1/tgg2*, but not in *pen2*. This differs from age-related developmental senescence in which early senescence was not observed in the *tgg1/tgg2* double mutant or in *pen2*.

The single *pen3* mutant was reported to display a subtle early senescence phenotype that was more obvious in high light (900 µmol photons m^−2^ s^−1^) and depended on the production and perception of SA (Stein et al., 2006). In addition, the single *pen1* mutant showed elevated SA in response to pathogens (Zhang et al., 2007). These observations suggest that PEN1 and PEN3 keep H_2_O_2_ and SA levels in check and prevent early senescence. We did not see an early senescence phenotype in the *pen3-1* or *pen3-3* alleles under our growth conditions; however, the *pen1/pen3* early senescence phenotype was obvious (**Supplemental Figure 5**), and depended on SA biosynthesis (**Figure 7**).

Overall, *cyp79B2/cyp79B3* mutants and *pen1/pen3* double mutants display early age-related developmental senescence, which, in the case of *pen1/pen3*, is mediated by SA. Neither lines show accelerated dark-induced detached leaf senescence. Our genetic analysis rules out the IAN pathway, and although reduction in free IAA is observed, there is no evidence in the extensive auxin literature that suggest a 50% reduction in free IAA results in early leaf senescence. It is possible that exosomes, replete with IG catabolites, are playing a protective role in leaf senescence. IAOx biosynthesis mutants cannot produce IGs, and *pen1/pen3* mutants are unlikely to be capable of producing exosomes.

## Materials and Methods

### Plant Genotypes and Growth Conditions

*Arabidopsis thaliana* Col-0 ecotype seeds and T-DNA insertion lines were obtained from the Arabidopsis Biological Resource Center (ABRC, Columbus, OH) and No-0 lines were obtained from RIKEN Bioresources. The *pen* double and triple mutants were gifts from Mats Andersson (Johansson et al., 2014), and *tgg1/tgg2* was a gift from Qiaomei Wang who obtained the double mutant from Dr. George Jander. *pad3* (SALK_026585) and *cyp71A12/cyp71A13* were obtained from Erich Glawischnig (Müller et al., 2015). *cyp79B2* [At4g39950, SALK_130570, T-DNA insertion located 1512 bp downstream from the start codon in exon 1 and identical to *cyp79B2-1* used by Zhao et al. (2002)] and *cyp79B3* (At2g22330, GABI_198F06, T-DNA insertion located 1711 bp downstream of the start codon in exon 2) homozygous mutants were cross-fertilized to produce the *cyp79B2/cyb79B3* double mutant. **Supplemental Table 3** lists the T-DNA insertions shown in **Supplemental Figure 7** as well as primers used to amplify cDNA. For all mutants shown, primers amplified WT cDNA, but did not amplify respective mutant cDNA. The *sid2-1* mutant was obtained from the Arabidopsis Biological Resource Center (Nawrath and Métraux, 1999).

Seeds were sown on Sunshine Mix LC1 soil (Sun Gro Horticulture Distribution Inc., Bellevue, WA) pre-treated with 300 mg per gallon Gnatrol (Valent Inc., Walnut Creek, CA) to kill fungus gnat larvae. Seeds were cold shocked at 4°C for 72 h and then grown under long-day conditions (20 h of light at 34 µmol photons m^−2^ s^−1^, 24°C and 4 h of dark, 24°C) in a PERCIVAL INTELLUS growth chamber. Seeds were sown in a random arrangement in six-pack pots. Pots were rotated every 1–2 days to minimize effects of variability within the chamber. Plants were fertilized by sub-irrigation twice weekly with 1 ml/L of GRO POWER 4-8-2 (Gro-Power Inc., Chino, CA). Gnatrol was reapplied to the surface of the soil at each watering. Harvested leaves were marked with loosely tied thread around the petioles shortly after they emerged.

### Leaf Harvesting

Plants harvested at 28 days contained green leaves and 1- to 7-cm primary bolts with flowers beginning to develop. At 35 days, primary bolts had elongated and secondary bolts had formed. Leaves harvested at 42 days represented the beginning stages of senescence when siliques were present on primary and secondary bolts. Leaves harvested at 49 and 56 days represented senescent plants in which older rosette leaves showed signs of yellowing along the edges and siliques began to dry and brown. To account for circadian influences on transcription levels, leaves 6 and 7 utilized for chlorophyll, protein, and RNA experiments were harvested at approximately the same time (∼10:00 am). Leaves harvested for hydrogen peroxide detection experiments were harvested between 6:00 and 8:00 am. Tissue for dark-induced senescence was harvested from leaf 5 at 21 days. One leaf disk was collected from each leaf using a ¼-inch-diameter hole punch, floated on water in a petri dish, and placed in a dark cabinet. Disks were removed at 0, 2, 4, and 7 days and frozen in liquid nitrogen in a 1.5-ml tube and stored at −80°C. All experiments were performed at least three times with similar results, with the exception of the fitness experiment shown in **Supplemental Figure 4**, which grew for 85 days and was performed once. Data presented in figures are from one biological replicate, with the number of samples (*n*) indicated.

### Quantitative Real-Time PCR

Total RNA was extracted from leaves using Trizol^™^ reagent following the manufacturer’s protocol with the addition of one ethanol precipitation after the standard isopropanol precipitation. The second precipitation removed trace phenol. qRT-PCR with SYBR green was performed in an Applied Biosystems StepOnePlus^™^ or a QuantStudio 6 Real-Time PCR machine (Life Technologies, Inc., Carlsbad, CA) to measure expression levels of SURGs and SDRGs as described (Brusslan et al., 2012) with the exception of the ACT2 primer pair (ACT2_F 5’GCGACTTGACAGAGAAGAAC, ACT2_R 5’GAAAGAGCGGAAGAAGATGAG. Transcript abundance was calculated by subtracting ΔC_T_ [the ΔC_T_ is calculated as the C_T_ value of gene of interest – C_T_ value of ACT2 (Livak and Schmittgen, 2001)] from 40. Other real-time primers used for the first time in this study are listed in **Supplemental Table 3**. The 40 − ΔC_T_ values were subject to one-way ANOVA and Sidak’s multiple comparison test to test for significant differences.

### Protein and Chlorophyll Extraction and Quantification

Two leaf disks were collected from leaf 6 (one for chlorophyll and one for protein) using a ¼-inch-diameter hole punch. Disks were frozen in liquid nitrogen in a 1.5-ml tube and stored at −80°C. Proteins were extracted and measured using the Bradford assay (Jones et al., 1989). Samples were ground in 100 μl of 0.1 M sodium hydroxide (NaOH) per leaf disk, incubated at room temperature for 30 min, and then centrifuged (14,000 × RPM) for 5 min. The supernatant was transferred to a fresh tube and stored at −20°C. A bovine serum albumin (BSA) standard curve ranging from 0 to 20 μg/ml was prepared. Soluble polyvinylpyrollidone (PVP) (3 mg/ml) was mixed with 1:4 diluted BioRad Protein Assay reagent (BioRad Inc., Hercules, CA). Triplicate samples (10 µl) and standards (0, 5, 10, 15, and 20 µg/ml) were plated on a 96-well plate, 200 μl of Bradford/PVP reagent was added to each well, and absorbance was read (OD_595_) after 15-min incubation at 25°C.

Disks used for chlorophyll measurements were incubated in 800 μl of dimethylformamide (DMF) in the dark overnight. Absorbance of samples (200 µl) and a DMF blank were read at 647 and 664 nm in a quartz microplate and used to calculate chlorophyll levels per leaf disk (Porra et al., 1989).

### Fitness and Development Evaluation

To detect differences in plant development and fitness, seed yield and age at which plants transitioned from vegetative to reproductive phases were recorded. To track developmental differences, the age of the plant (days) and the number of leaves that were produced at the time when bolts reached 1–3 cm was recorded in eight individuals of each genotype. Seeds were collected from the same plants continuously until 85 days, and the weight of 100 seeds was measured and used to calculate total seed production. One silique similar in size and relative position from the end of the bolt was also collected from each of these plants and dried, and the number of seeds within each silique was recorded. Bolts were removed and dried for 2 days at 50°C, and dry weight was recorded.

### IAA Content Analysis

IAA was measured by using GC-MS/MS by the Plant Metabolomics Facility at the University of Minnesota (Barkawi et al., 2010; Liu et al., 2012).

### Glucosinolate Content Analysis

Glucosinolates were extracted, separated, identified, and quantified as described (Chen et al., 2003; Pang et al., 2009; Mostafa et al., 2016).

## Data Availability Statement

All datasets generated for this study are included in the manuscript/**Supplementary Files**.

## Author Contributions

JB wrote the manuscript and designed the experiments, JB and RC prepared the figures. RC, MCV, NC, CJ and CR designed and carried out the experiments, IM, WK, SChh, and SChe performed the glucosinolate measurements, data analysis, and edited the manuscript.

## Funding

Research reported in this publication was supported by the National Institute of General Medical Sciences of the National Institutes of Health under Award Numbers R25GM071638 (NC, CJ and CR) and SCORE SC3GM113810 to JB. The content is solely the responsibility of the authors and does not necessarily represent the official views of the National Institutes of Health. Other sources of support are California State University, Long Beach Graduate Research Fellowship to RC, National Science Foundation MCB1158000 to SChe, and a visiting scholarship from the Egyptian government represented by the Egyptian Cultural and Educational Bureau at Washington, DC, to IM.

## Supplementary Material

Supplemental Figure 1 IG-related gene expression is stable during leaf senescence. Leaves 4 and 5 were harvested at the time of bolting (T0) or 12 days after bolting (T12). Significant differences for each transcript in comparison to T0 are shown. Errors are 95% confidence interval, *n* = 6.

Supplemental Figure 2 PCR amplification of cDNA from WT (w) and cyp79*B2/cyp79B3* lines [*b2/b3-1* (1) and *b2/b3-2* (2)]. PCR amplification of cDNA showed that double mutant cDNA could not be amplified by primers that flanked the T-DNA insertions: GABI_198F06 for *CYP79B3* and SALK_130570 for *CYP79B2*. Primers downstream of the T-DNA for *CYP79B2* did not amplify mutant cDNA while those downstream of the *CYP79B3* T-DNA could, suggesting a partial *CYP79B3* mRNA is made in the double mutant while *CYP79B2* mRNA is absent. Marker VI (Roche Life Sciences, Indianapolis, IN) is the molecular weight standard.

Supplemental Figure 3 DAB staining detecting H_2_O_2_ in representative leaves 6 and 7 in WT and *cyp79B2/cyp79B3*. **(A)** Brown precipitate in leaves after chlorophyll is cleared indicates the presence of H_2_O_2_. Dark staining at base of petiole is due to wounding when leaf is cut from the plant. **(B)** DAB staining quantified using Image J. Values represent percent leaf area stained with DAB *n* = 10.* represents significant difference from WT (one-way ANOVA, *p*< 0.05).

Supplemental Figure 4 Reduced fitness in double mutants. **(A)** Plants were grown and seeds were continuously collected up to 85 days, after which bolts and seeds were dried, and weighed. **(B** and **C)** *cyp79B2/cyp79B3* double mutants produce significantly smaller bolts and fewer seeds, *n* = 8 individual plants for each genotype. Error bars represent 95% confidence interval. * indicates significant difference from WT Col-0 (one-way ANOVA, Tukey’s multiple comparison test, ****p*< 0.001).

Supplemental Figure 5 Yellow leaves are visible for *pen1/pen3*, but not for WT or either *pen1* or *pen3* single mutants. **(A)** Plants were grown for 46 days and inflorescences were removed prior to photography. **(B)** Plants were grown for 42 days and photographed in the soil with inflorescences. All images were cropped from one single photo. The *pen1/pen3* double mutant is derived from the *pen1-1* and *pen3-1* alleles.

Supplemental Figure 6 Verification of mutants in camalexin synthesis. Top row: SALK_026585 (*pad3*) DNA cannot be amplified by the LP/RP genomic primers, but is amplified by the LBP-Salk/RP primers indicating it is homozygous for the T-DNA insertion (Nafisi et al., 2007). Middle row: The *cyp71A12/cyp71A13* double mutant cannot be amplified by the *CYP71A13* F/R primers, but is amplified by LBP-Salk/RP primers (SALK_105163). The *CYP17A12* allele harbors a 5-bp deletion that is not observed in WT (Müller et al., 2015). Bottom row: The *tgg1/tgg2* double mutant cDNA does amplify with control primers (first group), but does not amplify with TGG1 (second group) and TGG2-specific primers (third group), (Barth and Jander, 2006).

Supplemental Figure 7 Total aliphatic glucosinolates (AG) decrease in older leaves, but total IG levels do not change. Leaves 6 and 7 were harvested at 28 and 42 days. Significant differences are shown for each line between 28 and 42 days (one-way ANOVA, Sidak’s multiple comparison test).

Supplemental Figure 8 Early senescence is not observed in single IG synthesis mutants or in IG biosynthesis regulatory mutants. **(A)** The gel image shows cDNA from mutant lines amplified with primers that flank the insertion site. For each mutant, the first lane is mutant cDNA, the second is WT cDNA and the third lane is mutant cDNA amplified with a positive control primer. For most lines, the *myc2-1* primers were used as the positive control. For *myc2* alleles, the *myb51* primers were used. T-DNA insertion allele names, primer sequences and expected PCR product size are listed in Supplemental Table 3. For each mutant, a full-length mRNA was not produced. **(B)** Plants were grown for 32 days and leaf 3 was harvested for chlorophyll (*n* = 6, top panel) and leaf 4 was harvested for RNA (*n* = 3) and *NIT2* expression was quantified by real-time qPCR (bottom panel). The *pen1/pen3* double mutant was the only line to display early senescence. Significance values are shown for comparisons to WT (one-way ANOVA, Sidak’s multiple comparison test). Lines from the SALK and GABI collection were compared to WT-Col and RIKEN lines (Pst and Psh) and were compared to appropriate WT-NO lines obtained from RIKEN.

Supplemental Table 1 Growth and Development of *cyp79B2/cyp79B3* Double Mutants

Supplemental Table 2 Seed Yield and Bolt Weight in *cyp79B2/cyp79B3* Double Mutants
