# Supplementary figures and images for: Negative Regulation of Age-Related Developmental Leaf Senescence by the IAOx Pathway, PEN1, and PEN3
Source: Front Plant Sci. 2019 Oct 8;10:1202. doi: 10.3389/fpls.2019.01202 (PMC6792297; doi:10.3389/fpls.2019.01202)

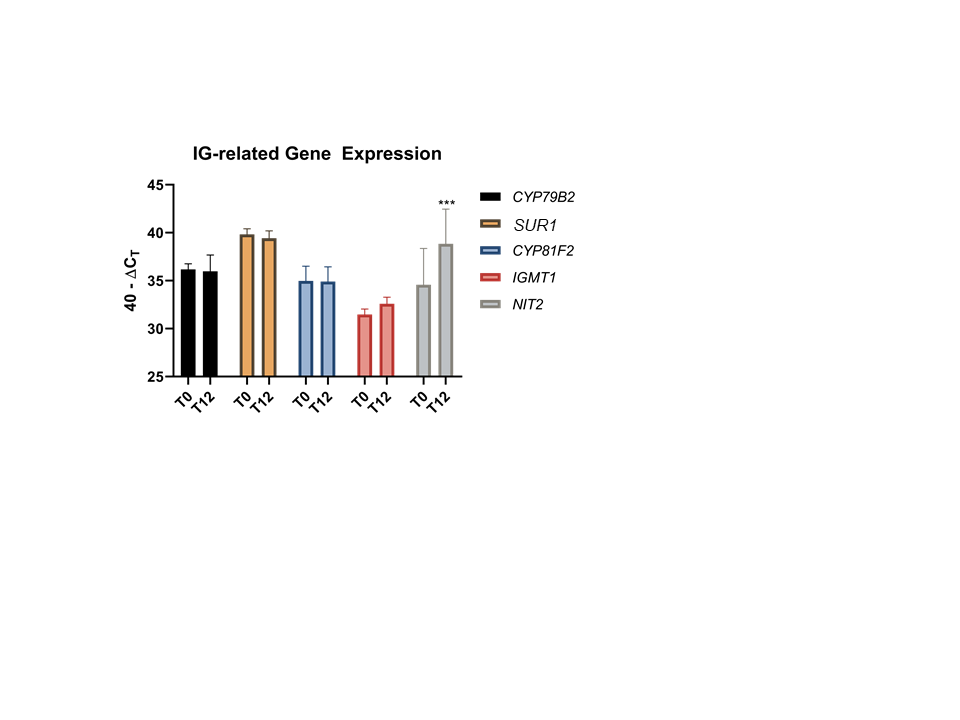

Supplement: Supplemental Figure 1 — IG-related gene expression is stable during leaf senescence. Leaves 4 and 5 were harvested at the time of bolting (T0) or 12 days after bolting (T12). Significant differences for each transcript in comparison to T0 are shown. Errors are 95% confidence interval, n = 6. [file Image_1.tif]

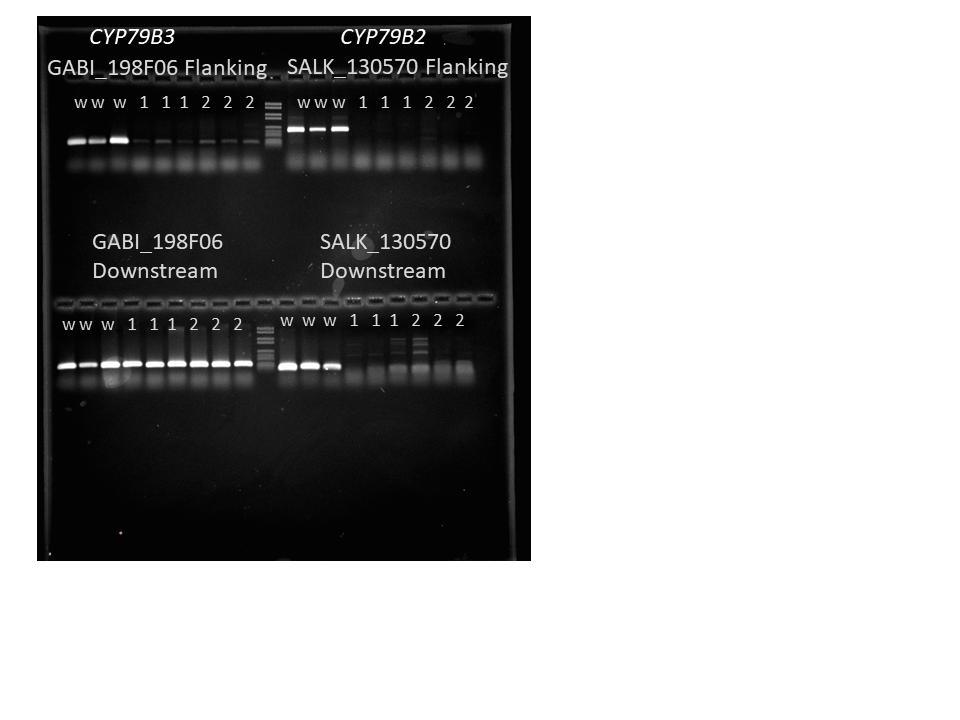

Supplement: Supplemental Figure 2 — PCR amplification of cDNA from WT (w) and cyp79B2/cyp79B3 lines [b2/b3-1 (1) and b2/b3-2 (2)]. PCR amplification of cDNA showed that double mutant cDNA could not be amplified by primers that flanked the T-DNA insertions: GABI_198F06 for CYP79B3 and SALK_130570 for CYP79B2. Primers downstream of the T-DNA for CYP79B2 did not amplify mutant cDNA while those downstream of the CYP79B3 T-DNA could, suggesting a partial CYP79B3 mRNA is made in the double mutant while CYP79B2 mRNA is absent. Marker VI (Roche Life Sciences, Indianapolis, IN) is the molecular weight standard. [file Image_2.tif]

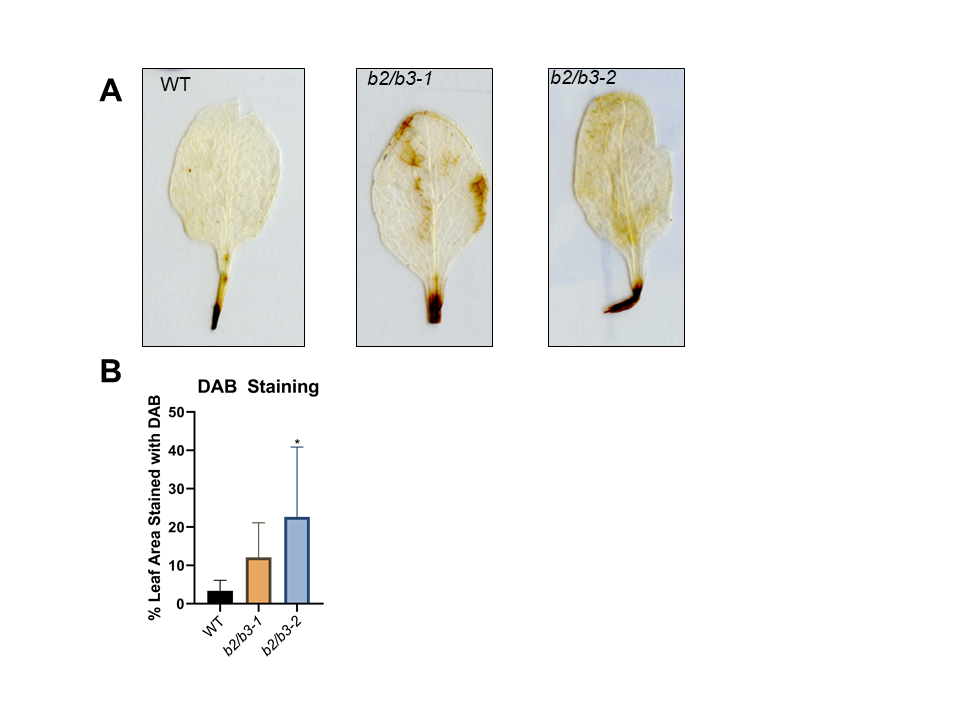

Supplement: Supplemental Figure 3 — DAB staining detecting H2O2 in representative leaves 6 and 7 in WT and cyp79B2/cyp79B3. (A) Brown precipitate in leaves after chlorophyll is cleared indicates the presence of H2O2. Dark staining at base of petiole is due to wounding when leaf is cut from the plant. (B) DAB staining quantified using Image J. Values represent percent leaf area stained with DAB n = 10.* represents significant difference from WT (one-way ANOVA, p< 0.05). [file Image_3.tif]

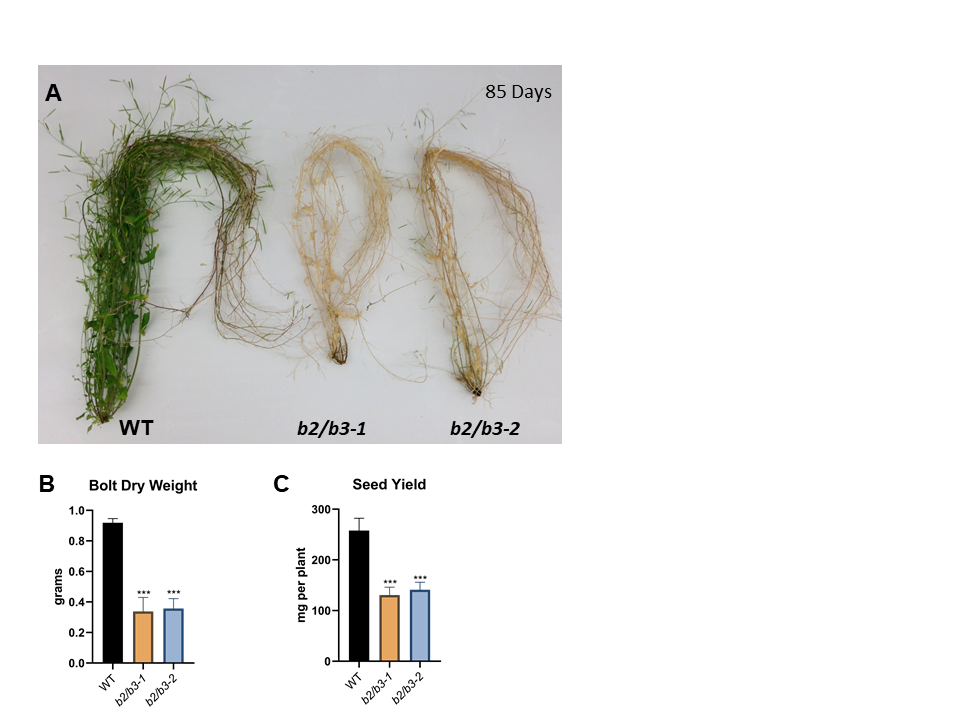

Supplement: Supplemental Figure 4 — Reduced fitness in double mutants. (A) Plants were grown and seeds were continuously collected up to 85 days, after which bolts and seeds were dried, and weighed. (B and C) cyp79B2/cyp79B3 double mutants produce significantly smaller bolts and fewer seeds, n = 8 individual plants for each genotype. Error bars represent 95% confidence interval. * indicates significant difference from WT Col-0 (one-way ANOVA, Tukey’s multiple comparison test, ***p< 0.001). [file Image_4.tif]

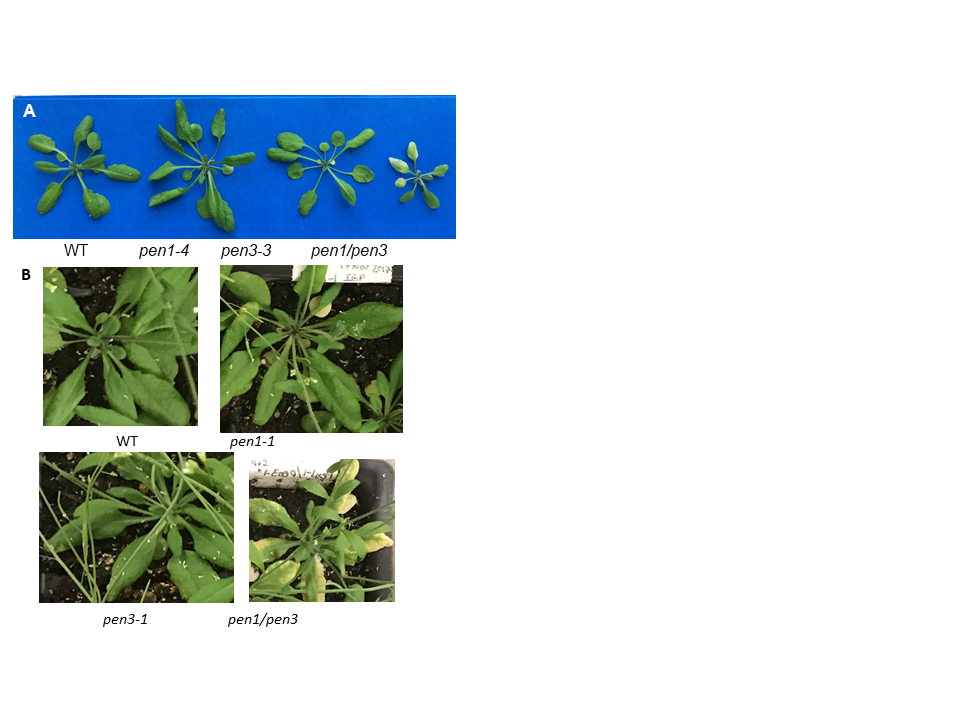

Supplement: Supplemental Figure 5 — Yellow leaves are visible for pen1/pen3, but not for WT or either pen1 or pen3 single mutants. (A) Plants were grown for 46 days and inflorescences were removed prior to photography. (B) Plants were grown for 42 days and photographed in the soil with inflorescences. All images were cropped from one single photo. The pen1/pen3 double mutant is derived from the pen1-1 and pen3-1 alleles. [file Image_5.tif]

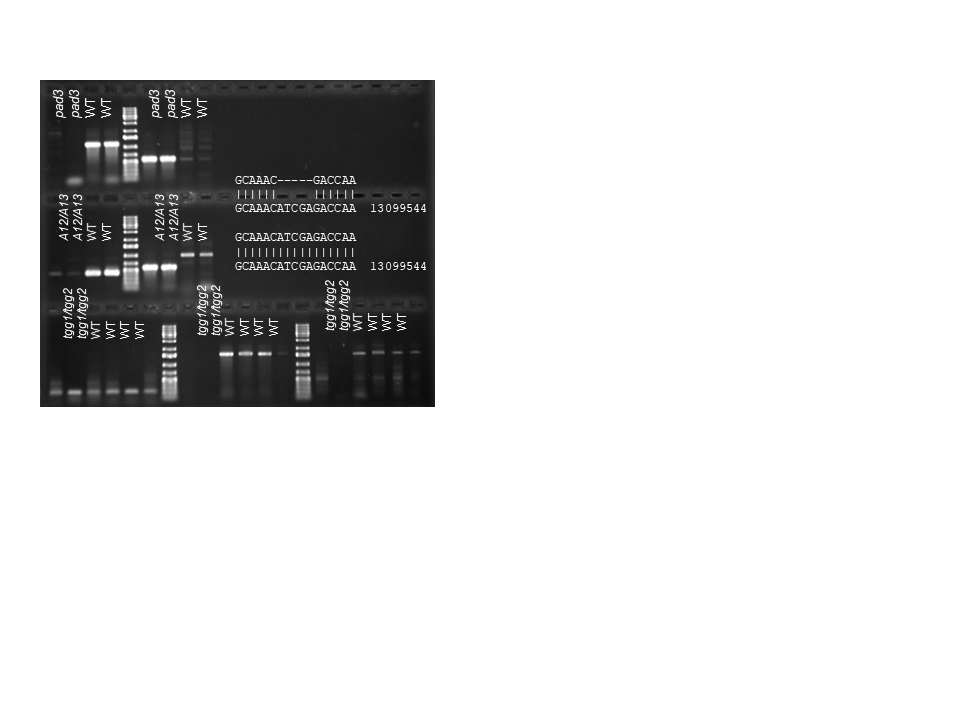

Supplement: Supplemental Figure 6 — Verification of mutants in camalexin synthesis. Top row: SALK_026585 (pad3) DNA cannot be amplified by the LP/RP genomic primers, but is amplified by the LBP-Salk/RP primers indicating it is homozygous for the T-DNA insertion (Nafisi et al., 2007). Middle row: The cyp71A12/cyp71A13 double mutant cannot be amplified by the CYP71A13 F/R primers, but is amplified by LBP-Salk/RP primers (SALK_105163). The CYP17A12 allele harbors a 5-bp deletion that is not observed in WT (Müller et al., 2015). Bottom row: The tgg1/tgg2 double mutant cDNA does amplify with control primers (first group), but does not amplify with TGG1 (second group) and TGG2-specific primers (third group), (Barth and Jander, 2006). [file Image_6.tif]

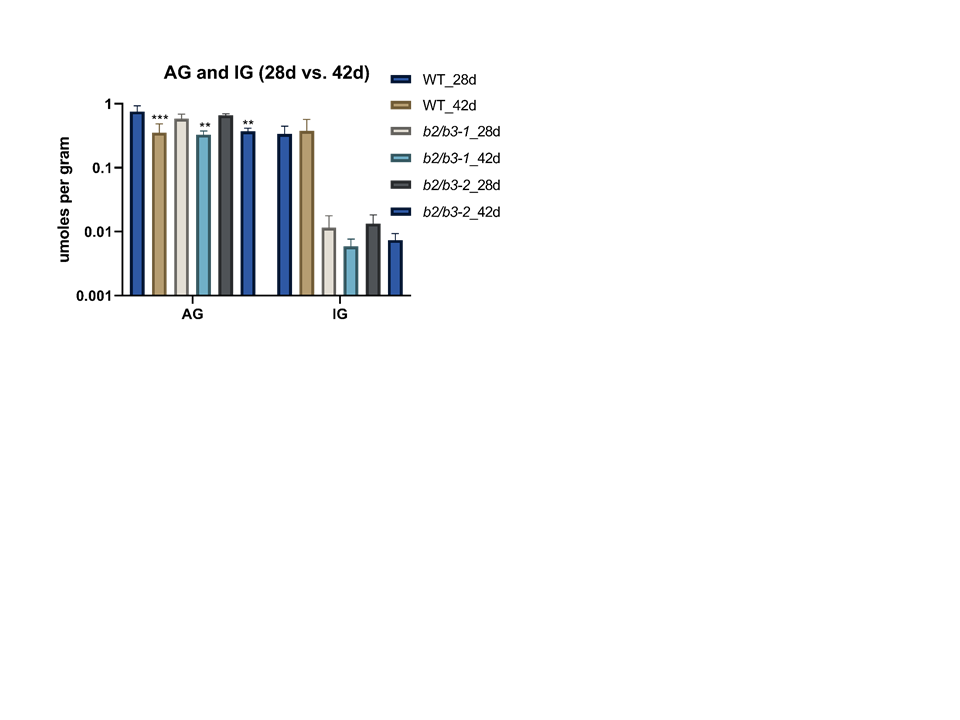

Supplement: Supplemental Figure 7 — Total aliphatic glucosinolates (AG) decrease in older leaves, but total IG levels do not change. Leaves 6 and 7 were harvested at 28 and 42 days. Significant differences are shown for each line between 28 and 42 days (one-way ANOVA, Sidak’s multiple comparison test). [file Image_7.tif]

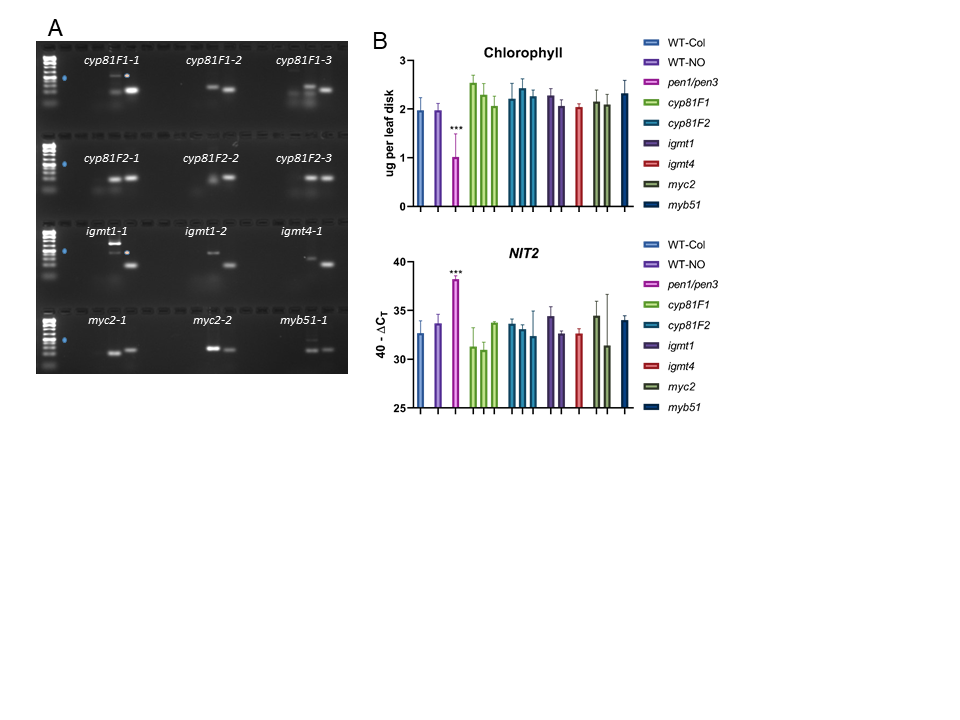

Supplement: Supplemental Figure 8 — Early senescence is not observed in single IG synthesis mutants or in IG biosynthesis regulatory mutants. (A) The gel image shows cDNA from mutant lines amplified with primers that flank the insertion site. For each mutant, the first lane is mutant cDNA, the second is WT cDNA and the third lane is mutant cDNA amplified with a positive control primer. For most lines, the myc2-1 primers were used as the positive control. For myc2 alleles, the myb51 primers were used. T-DNA insertion allele names, primer sequences and expected PCR product size are listed in Supplemental Table 3. For each mutant, a full-length mRNA was not produced. (B) Plants were grown for 32 days and leaf 3 was harvested for chlorophyll (n = 6, top panel) and leaf 4 was harvested for RNA (n = 3) and NIT2 expression was quantified by real-time qPCR (bottom panel). The pen1/pen3 double mutant was the only line to display early senescence. Significance values are shown for comparisons to WT (one-way ANOVA, Sidak’s multiple comparison test). Lines from the SALK and GABI collection were compared to WT-Col and RIKEN lines (Pst and Psh) and were compared to appropriate WT-NO lines obtained from RIKEN. [file Image_8.tif]
