# Supplementary material for: Negative Regulation of Age-Related Developmental Leaf Senescence by the IAOx Pathway, PEN1, and PEN3
Source: Front Plant Sci. 2019 Oct 8;10:1202. doi: 10.3389/fpls.2019.01202 (PMC6792297; doi:10.3389/fpls.2019.01202)
Supplement: Supplemental Table 1 — Growth and Development of cyp79B2/cyp79B3 Double Mutants [file Table_1.docx]

| Supplemental Table 1. Growth and Development of *cyp79B2/cyp79B3* Double Mutants | | | | |  |
| --- | --- | --- | --- | --- | --- |
|  | Age (Days) When Bolts | # Leaves When Bolts | Size Fully Expanded Leaf 6 | Size Fully Expanded Leaf 7 | |
|  | 1-3cm (n=8) | 1-3cm (n=8) | (cm^2^ n=5) | (cm^2^) n=5 | |
| Col-0 | 29.5 ± 0.64 | 12.25 ±0.49 | 2.318 ±0.23 | 2.687± 0.30 | |
| *b2/b3-1* | **26.75 ± 0.72***** | **9.88 ± 0.44 ***** | **1.938 ± 0.19 *** | **2.114 ± 0.37 *** | |
| *b2/b3-2* | **26.25 ± 0.49***** | **9.12 ± 2.4*** | **1.553 ± 0.38***** | **2.039 ± 0.21**** | |
| Values represent the mean ± 95% confidence interval. Values in bold are significantly different from Col-0. (Student's t-test, * p<0.05, ** p<0.01, *** p<0.001) | | | | |  |
