# Supplementary material for: Negative Regulation of Age-Related Developmental Leaf Senescence by the IAOx Pathway, PEN1, and PEN3
Source: Front Plant Sci. 2019 Oct 8;10:1202. doi: 10.3389/fpls.2019.01202 (PMC6792297; doi:10.3389/fpls.2019.01202)
Supplement: Supplemental Table 2 — Seed Yield and Bolt Weight in cyp79B2/cyp79B3 Double Mutants [file Table_2.docx]

| Supplemental Table 2. Seed Yield and Bolt Weight in *cyp79B2/cyp79B3* Double Mutants | | | | |  |  |  |
| --- | --- | --- | --- | --- | --- | --- | --- |
|  | Seed Yield | Seed | Hundred Seed | Seeds | Germination | Dry Weight of |  |
|  | (mg/plant) | Number/Plant | Weight (mg) | Per Silique | Rate | Bolts (g) |  |
| Col-0 | 257.9 ± 20.2 | 13927.9 ± 1087 | 1.86 ± 0.15 | 50.5 ± 3.4 | 0.972 ±0.003 | 0.9185 ± 0.02 |  |
| *b2/b3-1* | **130.5 ± 12.8***** | **7489.1 ± 917***** | 1.76 ± 0.16 | 45.8 ± 2.8 | 0.975 ±0.008 | **0.3375 ± 0.08***** |  |
| *b2/b3-2* | **141.2 ± 12.1***** | **7578.4 ± 950***** | 1.89 ± 0.13 | 50.9 ± 1.6 | 0.980±0.004 | **0.35706 ± 0.05***** |  |
| Values represent the mean ± 95% confidence interval. Values in bold are significantly different from wild-type. (Student's t-test, * p<0.05, ** p<0.01, *** p<0.001) n=8. | | | | | | | |
